# Use of machine learning techniques to identify risk factors for RV failure in LVAD patients

**DOI:** 10.3389/fcvm.2022.848789

**Published:** 2022-09-14

**Authors:** Nandini Nair

**Affiliations:** Division of Cardiology, Department of Medicine, Texas Tech Health Sciences Center (TTUHSC), Odessa, TX, United States

**Keywords:** left ventricular assist device (LVAD), machine learning, risk prediction models, RV failure, AI technology

## Introduction

With increasing use of Mechanical Circulatory Support (MCS) in the last decade and its evolution currently as a standard therapy for patients with end stage heart failure (HF), it is becoming imperative to derive better risk prediction models to improve outcomes. The evolution of MCS and the transition from the older pulsatile-assist devices to the newer continuous flow pumps have ushered in an era of benefits for the HF with reduced ejection fraction population (1–8). However, challenges still persist in post implantation management in the long and short terms. Risk prediction models impact patient selection and, in turn, post-implantation outcomes. One of the most important factors influencing morbidity and mortality in the patients with left ventricular assist device (LVAD) is right ventricular failure (RVF). RVF can occur in ~10–40% of cases, depending on the definition used to describe such failure (3–8). Several risk prediction models exist in the current literature, which predict RVF in patients with LVAD (9–16). This article attempts to address the need for improved risk prediction models using artificial intelligence (AI) technology.

## Impact of RV failure on outcomes in the LVAD population

RVF after LVAD implantation is a major cause of morbidity and mortality in this population. Hence, early recognition of risk factors and taking appropriate steps to prevent RVF remain safest options. RVF occurs due to pre-implantation clinical characteristics of the patient, as well as intraoperative/perioperative issues that occur during these periods.

Preoperative RV dysfunction is a major factor in patients with end-stage HF whether ischemic or non-ischemic. Predisposing factors include development of chronic secondary pulmonary hypertension, mitral regurgitation (MR), and primary disease of the myocardium. Several parameters determined by invasive hemodynamic parameters from preoperative right heart catheterization, such as low RV stroke work index (RVSWI), central venous pressure (CVP) to the pulmonary capillary wedge pressure (PCWP) ratio, with a ratio >0.63 associated with RV failure and pulmonary arterial pulsatility index (PAPi), are valuable in predicting RVF (11, 14, 17). Additionally, echocardiographic parameters, such as RA/RV size, the RV/LV ratio, left atrium volume index (LAVI), tricuspid annular plane systolic excursion (TAPSE), RV global free wall strain, and RV fractional area change (RVFAC), have all been used to predict RVF and tricuspid regurgitation severity (18).

RVF post LVAD implantation can be classified as acute (<48 h), early (>48 h–<14 days) and late (>14 days). *De novo* RV dysfunction can develop after LVAD implantation, while mild to moderate preoperative RV dysfunction can progress to frank RV failure due to intraoperative and perioperative factors. Cardioplegia leading to relative stunning of the RV myocardium has been noted. Cardiopulmonary bypass can itself initiate cytokine release, systemic inflammatory response syndrome (SIRS), and increased pulmonary vascular resistance (PVR) resulting in RVF.

Additionally, intraoperatively, myocardial ischemia, air embolism, mechanical compression of the PA, tamponade, and impact of LVAD circulation can result in RVF. Increased flow from the LV/LVAD and the consequent increase in venous return to the RV lead to increased RV preload. Loss of the septal contribution to overall RV function with paradoxical septal motion post LVAD implant can contribute to RVF. Despite the decrease in afterload post LVAD implant, the CVP/PCWP ratio worsens early after LVAD due to poor early RV adaptation, which progressively improves with time. Increased venous return to the RV due to rapid stepping up of LVAD speed leads to bulging of the interventricular septum into the LV, causing RV dilation and, therefore, worsening tricuspid regurgitation. Acute hypoxemia and resultant pulmonary vasoconstriction, worsening PVR, will cause RVF. Acute renal failure with increased CVP and metabolic and/or respiratory acidosis contribute to RVF. Increased risk of perioperative bleeding secondary to redo sternotomy and transfusion has been associated with SIRS and worsening RV function. Sustained atrial and ventricular tachyarrhythmias deteriorate RV function in addition.

RVF treatment should include cautious optimization of LVAD speed, diuresis/ultrafiltration, and volume optimization. Inhaled nitric oxide should be provided for pulmonary hypertension and increased RVR or use oral phosphodiesterase-5 inhibitors. Arrythmias should be treated, and, if RVF still persists despite medical management, mechanical support should be provided (RVAD/ECMO). Late RV failure can occur in the presence or absence of normal LVAD function and is difficult to treat with poor long-term outcomes. Body mass index (BMI) >29, BUN >41, and diabetes mellitus were significant predictors of late RVF. Late RVF is associated with worse 5-year posttransplant survival compared with patients who did not develop RVF (19).

LVADs may be designed for long-term hemodynamic support, but RVF still remains a challenge in >30% of patients in the early post LVAD period. RVF causes a significant increase in morbidity and mortality, whether they are bridged to transplantation or are on it as destination therapy. Therefore, RVF should be prevented by robust patient selection using appropriate preoperative risk prediction tools to identify the best LVAD candidates and by efficient perioperative management. Early diagnosis of RVF is the key to improving outcomes. There is, therefore, a need for identifying early predictors of RVF and further refinement of treatment strategies to achieve better outcomes (20).

The effect of RV failure on LVAD outcomes includes increased mortality, deteriorating renal function and longer length of stay in the ICU, all of which contribute to increased morbidity in addition to its effects on mortality (21).

## Current risk prediction scores and their limitations

There are a number of risk prediction scores at the present time, all of which have their advantages and limitations. The earliest of the models, which has been the Michigan RVF risk score put forth in 2008, was a single-center retrospective study. Other models have been compared to this. It was the most validated at 16, with a median c-statistic of 0.61. It used 4 binary pre-LVAD clinical variables. There was a higher concern for risk of bias due to variable RVF definition in the validation studies and indication bias due to inclusion of planned BIVAD patients and overfitting resulting in low RVF rates (8, 9).

The EUROMACS model was similarly a retrospective study involving multiple centers using five binary variables for early RVF. It was validated 5 times with a median c-statistic of 0.65. In this model, risk of bias was uncertain because the validation studies had variable definitions of RVF. Registry data were used, which had the inherent problem of missing data. The size of the cohort in the derivation study was large; hence, the applicability concern was low (8, 10).

The Penn model put forth in 2008 was a retrospective single-center study for severe early RVF and used 6 binary variables. It was validated 5 times with a median c-statistic of 0.63. Patients with planned BIVAD resulted in indication bias. RVF definitions varied and the study was impacted by missing data and low RVF risk patients being excluded (8, 11).

The Utah model was a single-center, retrospective study, with eight categorical variables for early RVF. It was validated seven times with a median c-statistic of 0.55. The variables were overfitted, inadequately powered; patients with missing data, selection bias, and varied RVF definitions were problems with this model too (8, 12).

The CRITT model, which is also a single-center, retrospective study, used central venous pressure >15 mm Hg, severe RV dysfunction, pre-op mechanical ventilation/intubation, severe tricuspid regurgitation, and tachycardia for predicting risk for RVF. It was validated 5 times with a median c-statistic of 0.63. It was a single-center retrospective study. This model had indication bias due to inclusion of planned BIVADs in the derivation study. Applicability was a concern due to large number of pulsatile LVADs in the derivation batch and non-uniform RVF definitions in the validation batches (8, 13).

The model put forth by Kormos et al. used 3 binary variables to predict early RVF. It was validated five times with a median c-statistic of 0.61. This model was derived from a *post-hoc* analysis of a cohort of belonging to the multicenter HeartMate II trial, and, hence, universal applicability was a concern. The model exhibited an inclusion bias because it only included a highly selected population who were all bridged to transplantation. This model had variable RVF definitions and lacked adequate power for analysis (8, 14).

The Pittsburgh Decision Tree uses AI. It was a single-center, retrospective study. It used eight binary variables for early severe RVF. It was validated two times with a median c-statistic of 0.53. Variable RVF definitions and low RVF rates and overfitting were all noted in this model (8, 15).

Overall multiple limitations were noted in all of the existing risk models, making them difficult to be universally applicable for RVF. The definitions for RVF used were highly varied. The percentage of continuous flow pumps was a variable in the different studies, making it less predictable for the present day as pulsatile LVADs have essentially phased out. Not all models reported calibration. Validation groups appear to have not been stringent in patient selection or RVF definition, making them less reliable. Additionally, the type of RVF predicted whether acute, early or late was highly varied. This leads to heterogeneity, depending on the variability from institution to institution of medical vs. device therapies for RVF. Additionally, the existing models have lower-than-ideal c-statistics, ranging from 0.55 to 0.65 (8–15).

## Utilizing AI technology to predict risk

Use of machine learning (ML) in developing risk scores for HF mortality seems to have an edge over conventional methods and is currently looking encouraging. The MARKER-HF score has a c-statistic of 0.88 and has been validated in 2 external study cohorts. This model used a boosted decision tree algorithm to derive a model based on automated training using two well-defined cohorts—the low and high groups (22). In another study, telemetry data analyses from a wearable monitor used a general machine learning similarity-based modeling, which was used to predict HF hospitalization. Receiver operating characteristic curves showed a c-statistic of 0.86–89 using the analytics platform. The alert from such prediction models could help clinicians intervene before an HF hospitalization occurs (23). Prediction of mortality post LVAD implantation, in general, has been attempted using Bayesian network analysis with a c-statistic of 0.7 for 1-, 3-, and 12-month mortality (24).

Applications of machine learning algorithms to assess tricuspid annulus excursion on 2-dimensional (2D) and 3-dimensional (3D) echocardiography have been attempted with considerable success in assessment of RV function. Application of an automated segmented model based on neural network architecture was used in a 2D echo image analysis. An ML algorithm was trained and tested in a 6-fold cross validation approach. Tricuspid annular displacement measurements using manual and automated ML segmentation showed that the automated approach was comparable to MRI data. The ROC curves used to test the model showed a c-statistic of 0.69–0.73 in a small population studied. The ML-driven assessment used a deep learning framework and was time efficient with a processing time of <1 s (25). In another study using ML-based algorithms using 3D echocardiographic images, RV volumes and ejection fraction measurements were made with excellent reproducibility, suggesting that automated analysis of data may be more efficient (26).

A Bayesian network analysis-driven model for acute, early, and late RVF post LVAD implantation published in 2016 was based on the INTERMACS registry. The acute, early, and late RVF models comprised of 33, 34, and 33 preoperative variables (from demographics, hemodynamics, laboratory values, and medications), respectively. The performance of this model was superior to earlier models (c-statistic of 0.53–0.65) with c-statistics of 0.9, 0.83, and 0.88 for acute, early, and late RVF, respectively (27). The study had limitations, such as missing data, which are inherent to registry data.

**Figure 1:**
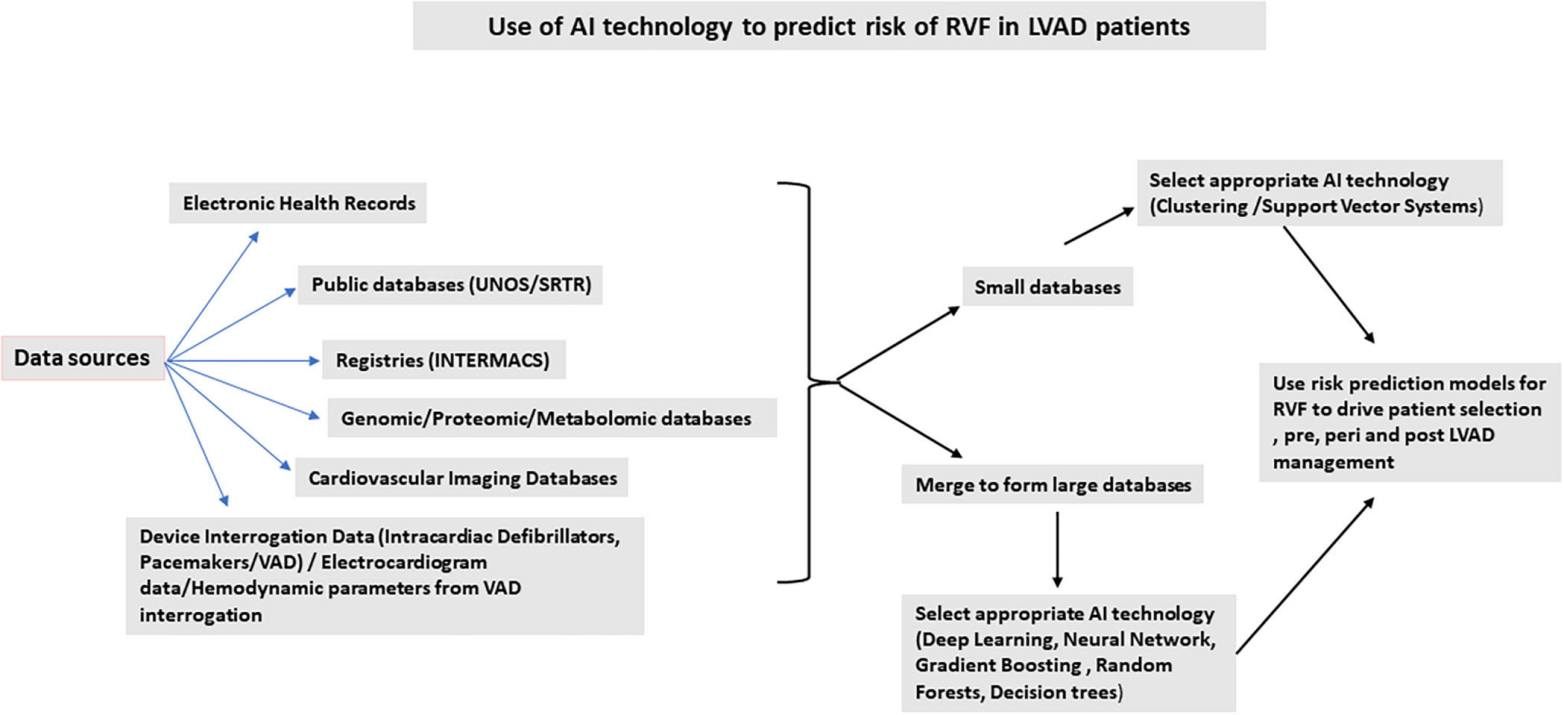
Use of AI technology to predict risk of right ventricular failure in left ventricular assist device patients.

Figure 1 summarizes possible applications of AI technology to develop risk prediction models for RV dysfunction in the LVAD population. Risk prediction helps with improving outcomes if applied to patient selection and management pre-, peri-, and post-device implantation. Incorporating hemodynamic parameters from invasive hemodynamics as well as LVAD parameters and speed can predict RV failure. In a small, single-center study, a new hemodynamic index generated using mean arterial pressure, the ratio of pulmonary artery wedge pressure and central venous pressure, and the ratio of the set pump speed to maximum pump speed in a ramp study showed that this index can be used to predict RV failure. A c-statistic derived from the Area Under the Curve using a Receiver Operative Curve was high at 0.86 (28). A combination of clinical and hemodynamic parameters can be used to generate better and robust risk models. Large databases maybe generated by data pooling from different smaller databases. AI technology can then be applied following data generation and normalization. The AI technology can then be used to generate risk models for patient selection pre-LVAD, a bridge to transplant and destination populations. The type of AI technology used will depend on the type of database used for analysis. The pros and cons for each different type of AI technology should be considered for each individual analysis undertaken, depending on the size of the database and its tendency to overfit data, time factor, and ease of interpretation. Considering the impact of RVF on post-LVAD outcomes, risk stratification for RV failure is one of the major determinants for patient survival and becomes the most important strategy to improve patient outcomes (29–31).

In summary, the four major aspects of machine learning are collection of data, building an appropriate mathematical model, constructing a learning algorithm, and defining at the final model for decision. Large data sets are ideal, especially for deep learning, which can be derived by combining smaller datasets. The right mathematical model should precisely represent the data and key properties of the problem in question. To achieve best predictive performance flexible models such as those based on the deep neural networks or the Gaussian process should be used. The learning algorithm is then used for computation of variables inherent in the model using the data set. The final process is building the algorithms for precise prediction (32–34).

## Discussion

In summary, this review shows that existing literature points to increased efficiency in data analysis and developing risk models using AI technologies. The performance of these models appears to be better than those developed using conventional systems in the present studies, which are largely retrospective. However, testing in larger prospective longitudinal populations still remains to be proven.

Models for prediction of RVF generated by conventional methods have limitations mainly due to universal assumptions of linearity. Regression models are simple and easy to perform as well as understand, but their use in model prediction is not as efficient as ML-based methods. ML methods are based on unbiased classification/clustering of attributes in the setting of a decision tree, neural network or algorithm. Hence, ML technology needs to be used in the setting of a balance between minimal training errors, especially as the models get more complex and its universal applicability. Additionally, recognition of important covariates to be used as input data is another major part of successful generation of risk prediction models. Improving interpretability of machine learning models of prediction is another area to consider.

## Future directions and challenges

Standardization of clinical behavior and accuracy of data collection remains a challenge. Algorithms for guideline-derived medical therapy vary across the globe, making it difficult to standardize the data collection. Prospective collection of data is, definitely, a requirement to generate large databases. Generation of large prospective databases will be the crux of generating robust risk prediction models, and validation of results using independent data sets will possibly help develop better risk prediction models. Developments of novel technologies for acquisition of bio signals and biosensors and secure data transmission could help generate more robust prospective databases contributing toward standardization of input and output variables. Finally, a multimodal approach to data collection will be more powerful in developing risk prediction models. Using powerful risk prediction models will open up new ways of approaching diagnosis and treatment in diverse subpopulations representing different races and socioeconomic strata.

## Author contributions

NN is responsible for the entire generation of the manuscript.

## Conflict of interest

The author declares that the research was conducted in the absence of any commercial or financial relationships that could be construed as a potential conflict of interest.

## Publisher's note

All claims expressed in this article are solely those of the authors and do not necessarily represent those of their affiliated organizations, or those of the publisher, the editors and the reviewers. Any product that may be evaluated in this article, or claim that may be made by its manufacturer, is not guaranteed or endorsed by the publisher.
